# Distribution of the CM-Dil-Labeled Human Umbilical Cord Vein Mesenchymal Stem Cells Migrated to the Cyclophosphamide-Injured Ovaries in C57BL/6 Mice

**DOI:** 10.29252/.23.3.200

**Published:** 2019-05

**Authors:** Ladan Jalalie, Mohammad Jafar Rezaie, Ali Jalili, Mohammad Ali Rezaee, Zakaria Vahabzadeh, Mohammad Reza Rahmani, Mojtaba Karimipoor, Mohammad Saeed Hakhamaneshi

**Affiliations:** 1 Student Research Committee, Kurdistan University of Medical Sciences, Sanandaj, Iran; 2 Cellular and Molecular Research Center, Research Institute for Health Development, Kurdistan University of Medical Sciences, Sanandaj, Iran; 3 Department of Anatomical Sciences, Faculty of Medicine, Kurdistan University of Medical Sciences, Sanandaj, Iran; 4 Cancer and Immunology Research Center, Kurdistan University of Medical Sciences, Sanandaj, Iran; 5 Zoonoses Research center, Research Institute for Health Development, Kurdistan University of Medical Sciences, Sanandaj, Iran; 6 Department of Medical Laboratory Sciences, Faculty of Paramedical, Kurdistan University of Medical Sciences, Sanandaj, Iran; 7 Liver and Digestive Research Center, Research Institute for Health Development, Kurdistan University of Medical Sciences, Sanandaj, Iran; 8 Department of Clinical Biochemistry, Faculty of Medicine, Kurdistan University of Medical Sciences, Sanandaj, Iran; 9 Department of Anatomy, Faculty of Medicine, Urmia University of Medical Sciences, Urmia, Iran; 10 Cellular and Molecular Research Center, Faculty of Medicine, Urmia University of Medical Sciences, Urmia, Iran

**Keywords:** Cyclophosphamide, Mesenchymal stem cells, Premature ovarian failure, Transplantation

## Abstract

**Background:**

Mesenchymal stem cells (MSCs) can be used to treat premature ovarian failure (POF). Different methods have already been applied to detect MSCs in tissues. This study aimed to investigate the quantitative distribution of CM-DiI-labeled human umbilical cord vein MSCs (hUCV-MSCs) in different regions of the ovarian tissue of the cyclophosphamide (CTX)-induced POF in mice.

**Methods:**

Adult female C57BL/6 mice (n = 40) were divided into four groups: (1) Mice receiving PBS as control (Ctrl) group; (2) mice receiving hUCV-MSCs intravenously as Ctrl + hUCV-MSCs group; (3) mice receiving CTX intraperitoneally (i.p.) as CTX group; (4) mice receiving CM-DiI-labeled hUCV-MSCs after CTX injection as CTX + hUCV-MSCs group. Histological changes and CM-DiI-labeled hUCV-MSCs distribution were analyzed in the ovarian tissues. Quantitative real-time PCR was performed to detect human mitochondrial cytochrome b (MTCYB) gene in the ovarian tissues of the mice.

**Results:**

The mean number of the fluorescent hUCV-MSCs was 20 ± 2.5 (57.1%) in the medulla, 11.3 ± 2.8 (32.2%) in the cortex, and 5.5 ± 1 (15%) in the germinal epithelium of the ovarian tissue (*p* < 0.05). Moreover, MTCYB gene was detected in the mice ovaries of the CTX + hUCV-MSCs group, but not in other groups.

**Conclusion:**

Our findings suggest that the distribution of the transplanted hUCV-MSCs in different regions of the ovarian tissue is not equal, and it is greater in the medulla than the cortex and germinal epithelium. This is the first report of quantitative distribution of MSCs in different regions of ovarian tissue in the POF model.

## INTRODUCTION

Premature ovarian failure (POF) is a heterogeneous syndrome in which menopause occurs before the age of 40. This failure is caused by a variety of genetic diseases such as fragile X syndrome and Turner syndrome, as well as by some autosomal disorders or medical interventions, including radiotherapy and chemotherapy[1,2]. POF is characterized by the decreased number of follicles, increased FSH levels, decreased estrogen, and amenorrhea[3,4].

Cyclophosphamide (CTX) is an alkylated chemotherapy drug that can cause POF and degenerative changes in ovarian tissue[5]. The goal of POF treatment is to increase the chance of fertility. Hormone replacement therapy has been used to treat POF, but it has systemic and cardiovascular complications[6]. In recent years, cell therapy has been proposed as a strategy for POF treatment with minimal side effects and considered in clinical practice[7,8].

Mesenchymal stem cells (MSCs) are highly important in regenerative medicine because of their inherent regenerative properties[9,10]. MSCs could increase the reproductive capacity of sterilized female animals in practical researches. Restorative effects of MSCs on injured ovaries have been observed in animal models of POF[7,11,12]. MSCs tracking and tissue distribution are interesting aspects of MSCs studies. Some fluorochrome materials have been used for labeling MSCs to be tracked in target tissues[13,14]. CM-DiI is a very effective and available lipophilic dyes binding to the membrane phospholipids[15,16]. It has low cytotoxicity for long-term culturing of labeled MSCs without significant effect on the cell properties[14,17].

Currently, MSCs have been known as a potential choice for the treatment of many diseases and tissue injuries. It is important to determine the distribution of MSCs in different parts of the target organ after administration of these stem cells. Although MSCs tracking has been conducted in previous studies[11,18], there is no report on quantitative distribution of MSCs in different regions of the ovarian tissue. This study aimed to investigate the quantitative distribution of labeled human umbilical cord vein MSCs (hUCV-MSCs) in different parts of the ovarian tissues in a mice model of CTX-induced POF.

## MATERIALS AND METHODS

### Animals and ethics

Female C57BL/6 mice (n = 40), at 7–8 weeks of age and weighing 25-30 g, were obtained from Animal House Center at the Kurdistan University of Medical Sciences (Sanandaj, Iran). The ethical approval of the study was obtained from the Animal Ethics Committee of the Kurdistan University of Medical Sciences in compliance with the guidelines published in the NIH (National Institutes of Health Publications) Guide for the Care and Use of Laboratory Animals (Ethic code: IR.MUK.REC.1395.185).

### Isolation and expansion of hUCV-MSCs

Following informed consents from the mothers, hUCV-MSCs were obtained from an operating room. Briefly, the umbilical cord veins were collected aseptically and transferred to the lab in sterile tubes on ice. After washing with Hank’s *buffered* salt solution (HBSS) containing 400 mg/L of KCl, 60 mg/L of KH_2_PO_4_, 100 mg/L of MgSO_4_-7H_2_O, 8 g/L of NaCl, 60 mg/L of Na_2_HPO_4_-2H_2_O, 1 g/L of Glucose, 140 mg/L of CaCl_2_, 100 mg /L of MgCl_2_-6H_2_O, 350 mg of NaHCO_3_, the veins were filled with collagenase IV (Gibco, USA) and incubated at 37 °C for 20 minutes. After centrifugation at 600 ×g for 15 minutes, the cells were removed, washed twice in sterile PBS and cultured in tissue culture flasks containing Dulbecco’s Modified Eagles Medium (DMEM; Gibco, USA) supplemented with 10% fetal bovine serum (Gibco, USA), 100 μg/ml of streptomycin, and 100U/ml of penicillin. The cultures were incubated at 37 °C in a humidified environment containing 5% CO_2_. After 48 hours, the non-adherent cells were discarded, and the media were replaced every three days. When the hUCV-MSCs reached 80-90% confluency, the adherent cells were trypsinized with 0.02% Trypsin (Sigma-Aldrich, USA), and then the cells were passaged up to passage 4[19].

### hUCV-MSCs morphology and immunophenotyping

hUCV-MSCs morphology was observed by an inverted microscope at passages 3-4. Moreover, the expression of hUCV-MSCs-related surface markers such as CD105 and CD73 and the lack of the CD45 and CD34 markers were evaluated by flow cytometry. In brief, after reaching 70-80% confluency, the cells were detached using Trypsin. The harvested cells were washed and resuspended in PBS. Aliquots of 1 × 10^6^ cells were incubated with FITC-labeled anti-CD45, anti-CD105, anti-CD34, and PE-labeled anti-CD73 in the dark at 4 °C for 30 minutes. After staining, the cells were fixed using paraformaldehyde, and the expression of the cell surface markers were detected using flow cytometry.

### CM-DiI-labeled hUCV-MSCs preparation

hUCV-MSCs were detached using Trypsin and resuspended at a concentration of 1 × 10^6^ cells/ml in HBSS buffer. CM-DiI dye stock was prepared as recommended by the manufacturer (Thermo Fisher Scientific Inc., Waltham, MA, USA). A confluent layer of 1 × 10^6^ MSCs/ml was stained using CM-DiI solution (5 µl) and was incubated at 37 °C for 15 minutes, then at 4 °C for 15 minutes. Next, the cell suspension was centrifuged, the media were removed, and the cells were washed twice in sterile PBS (pH 7.4). Subsequently, the labeled cells were cultured, maintained at sub-confluences and monitored for fluorescence using the Olympus BX51 microscope (Olympus, Tokyo, Japan).

### POF model induction, grouping, and hUCV-MSCs administration

To induce POF, mice were injected with 50 mg/kg CTX (Sigma-Aldrich, St. Louis, MO, USA) dissolved in PBS intraperitoneally (i.p.) for 15 consecutive days. The animals were divided into the following four groups, each group including 10 mice: (1) mice receiving PBS i.p. for 15 days and then 200 µl PBS via lateral tail vein as control (Ctrl) group; (2) mice receiving PBS i.p. for 15 days and then 1 × 10^6^ CM-DiI-labeled hUCV-MSCs in 200 µl PBS via lateral tail vein as Ctrl + hUCV-MSCs group; (3) mice receiving 50 mg/kg CTX i.p. for 15 consecutive days and then 200 µl PBS intravenously (i.v.) as CTX group; (4) mice receiving CTX for 15 consecutive days and then 1 × 10^6^ CM-DiI-labeled hUCV-MSCs as CTX + hUCV-MSCs group. One week after the hUCV-MSCs injection, the mice in all the study groups were euthanized by cervical dislocation, and their ovaries were removed under sterile conditions to conduct further experiments.

### Histological examination

Briefly, the ovaries of the mice were removed aseptically, washed with sterile PBS, fixed with 4% paraformaldehyde (Sigma, St. Louis, MO, USA), embedded in paraffin, serially sectioned at 5 µm thickness and then dehydrated using graded ethanol. Finally, the sections were stained with hematoxylin (Merck KGaA, Darmstadt, Germany) and eosin (H & E; Sigma, St. Louis, MO, USA).

### Quantification of labeled hUCV-MSCs

CM-DiI-labeled hUCV-MSCs were counted in 10 serial ovarian tissue sections (5 µm), in each mouse of the study groups[20]. The fluorescent cells were counted in different regions of the ovarian tissues with a fluorescent microscope (Olympus, Tokyo, Japan)[21]. The mean numbers of the CM-DiI-labeled hUCV-MSCs were calculated in the total ovarian tissue as well as in the medulla, cortex, and germinal epithelium of the ovary, separately. Also, the percentage of the counted cells in each region of the ovarian tissue was calculated compared to the total counted fluorescent cells in the ovarian tissue.

### DNA extraction and PCR

Real-time PCR was performed to detect the human mitochondrial cytochrome b (MTCYB) gene to confirm hUCV-MSCs migration to the target tissue. Mouse GAPDH gene was used as a reference for sample normalization and quantitative analysis in all the study groups. Specific primers were used to amplify the target genes. The forward and reverse primer sequences used for MTCYB were 5'-AGCCAC TTTCCACACAGAC-3' and 5'-AGTAGTATGGGAG TGGGAG-3', and for GAPDH included 5´-AATGTG TCCGTCGTGGATCTGA-3´ and 5´-GATGCCTGCT TCACCACCTTCT-3´, respectively. The amplicons predicted for MTCYB and GAPDH were 219 and 167 bp, respectively. DNA was extracted from 25 mg of the ovarian tissues in each group using QIAamp DNA Mini Kit (QIAGEN, Hilden, Germany) according to the manufacturer’s instructions. The concentration and purity of the DNA extracted from each sample were determined micro-spectrophoto-metrically (BioTek Instruments Inc., USA). The amplification and detection were performed using the Real Q Plus Master Mix Green without ROX™ (Ampliqon, Denmark) according to the protocols provided by manufacturer using a Rotor-Gene 6000 real-time PCR machine (Corbett Life Science, Sydney, Australia). Briefly, the PCR reaction was performed in a 25-μl final volume. Each reaction was composed of 12.5 μl of real-time PCR master mix, 7.5 μl of deionized water, 1 μl of each primer with a concentration of 10 μM, and 3 μl of the template DNA (30 ng/μl). Thermocycler thermal conditions included primary denaturation at 90 °C for 15 minutes, followed by 40 repetitive cycles at 90 °C for 60 seconds and then at 60 °C for 60 seconds. Relative copy number of human MTCYB and mouse GAPDH genes were calculated using the open access softwarbe LinRegPCR version 13[22].

### Statistical analysis

The statistical analyses of the data were conducted using SPSS 16 and one-way analysis of variance (one-way ANOVA). *p* value less than 0.05 was considered statistically significant.

## RESULTS

### hUCV-MSCs characterization

**Fig. 1:**
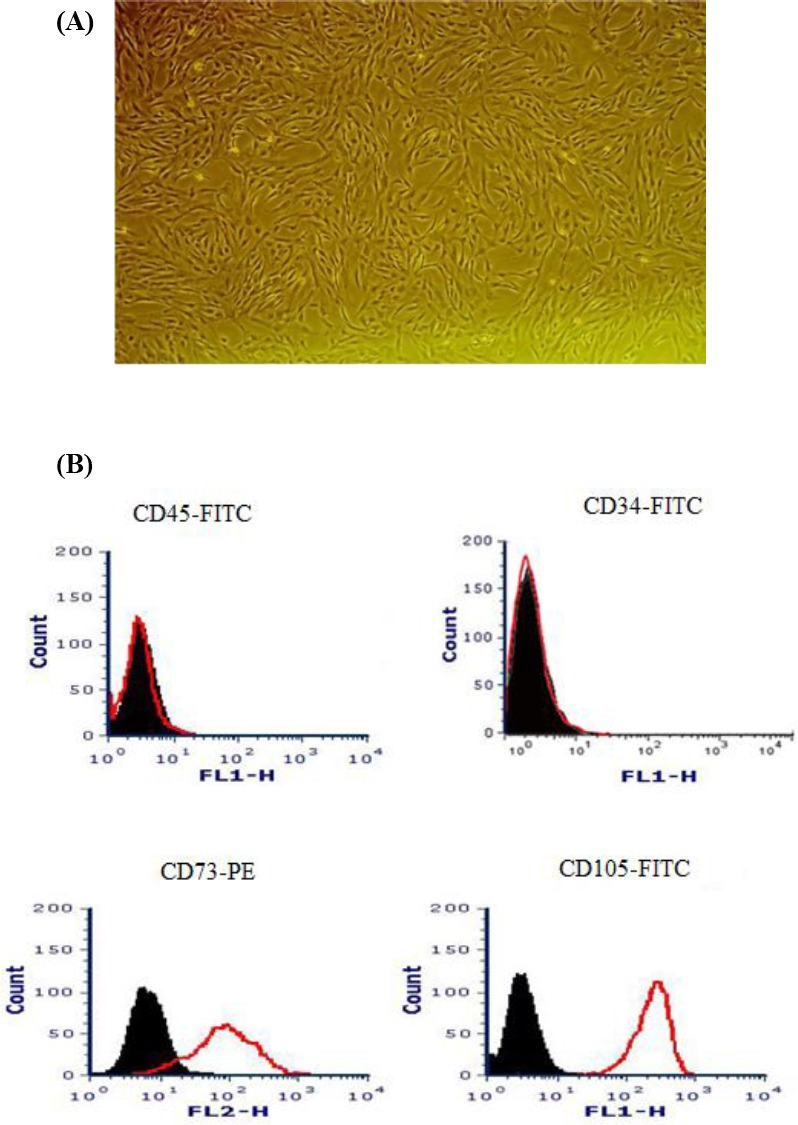
Human umbilical cord vein mesenchymal stem cells (hUCV-MSCs) morphology and immunophenotyping at passage 3. (A) The hUCV-MSCs showing an elongated and a fibroblast-like shape (magnification 100×); (B) hUCV-MSCs indicating to be positive for CD73 and CD105 and negative for the CD45 and CD34 surface markers.

Morphology of the hUCV-MSCs was examined under an inverted microscope. Colonies of the hUCV-MSCs were observed two days after the initial isolation. After a week, the surfaces of the cell culture flasks were filled, and spindle-shaped cells were observed in the passage 3 (Fig. 1A). Flow cytometry analysis indicated that hUCV-MSCs were positive for CD73 and CD105 and negative for CD45 and CD34 (Fig. 1B).

### Confirmation of POF after administration of CTX

**Fig. 2:**
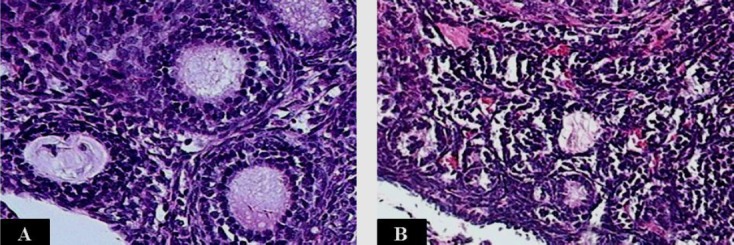
H & E staining of the mice ovarian tissue sections. (A) In the ovarian tissue section of the group receiving sterile PBS (Ctrl), normal growing follicles were observed. (B) In the ovarian tissue section of the mice receiving cyclophosphamide (CTX), healthy follicles decreased, and many atretic primordial and primary follicles were observed (magnification 200×).

The ovarian tissue sections of both CTX and Ctrl groups were used to evaluate the histological changes for the confirmation of POF induction. In mice receiving CTX, ovarian tissue sections stained with H & E showed a decrease in the number of the follicles, especially the primordial follicles. Also, many atretic follicles were observed in the stromal region of the CTX group (quantitative data are not shown; Fig. 2).

### Counting CM-DiI-labeled hUCV-MSCs

**Fig. 3:**
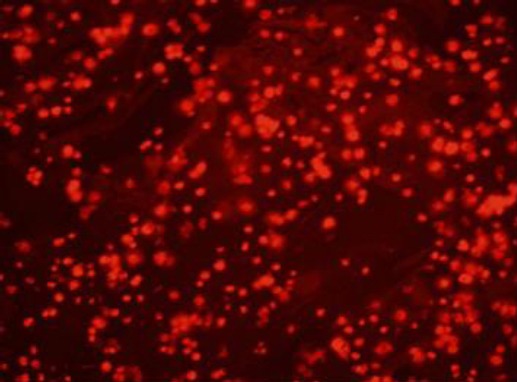
Photomicrograph of human umbilical cord vein mesenchymal stem cells (hUCV-MSCs) stained with CM-DiI fluorochrome. hUCV-MSCs were trypsinized at passage 3 and stained with CM-DiI *in vitro*, then the cells were observed under a fluorescence microscope (magnification 100×).

**Fig. 4:**
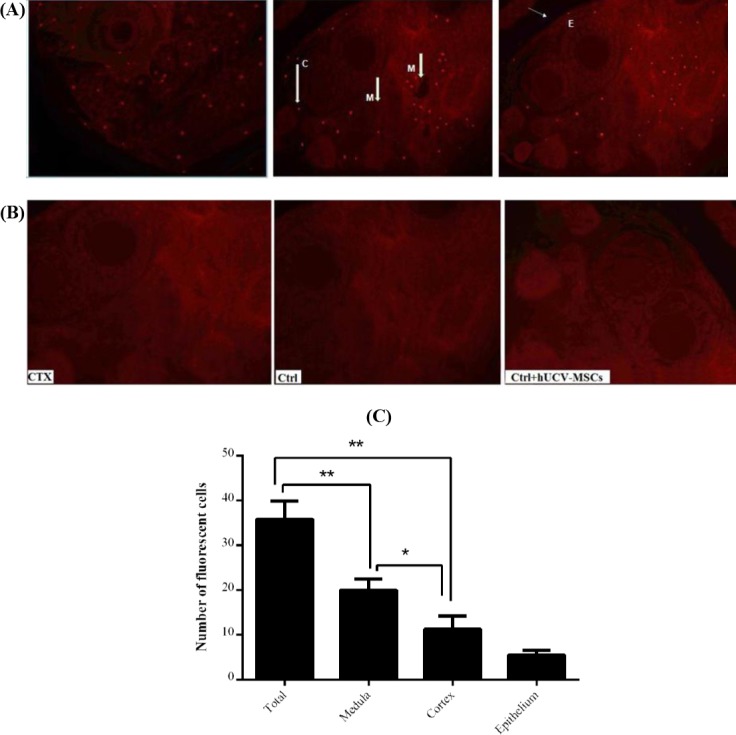
Red fluorescent CM-DiI-labeled human umbilical cord vein mesenchymal stem cells (hUCV-MSCs) detection in the mice ovarian regions. (A) Labeled hUCV-MSCs were detected in different regions of the ovarian tissue sections from CTX + hUCV-MSCs group; in the medulla (M) near the medullary veins, in the cortex (C), and in germinal epithelium (E); (B) fluorescent cells were not observed in the tissue sections of the CTX, Ctrl, and Ctrl + hUCV-MSCs groups; (C) the number of florescent hUCV-MSCs in different regions of the ovary in the CTX+ hUCV-MSC group was counted. The number of the labeled MSCs in the medulla is greater than the cortex and germinal epithelium (*p* < 0.001), and in the cortex, it is higher than germinal epithelium (*p* = 0.01). Data are shown as mean ± SD. ^*^p = 0.01, ^**^p < 0.001.

After staining with CM-DiI, hUCV-MSCs were observed using a fluorescence microscope at the magnification of 100 (Fig. 3). Subsequently, the ovarian tissue sections from the experimental groups were examined to observe the CM-DiI-labeled hUCV-MSCs using a fluorescent microscope. CM-DiI-labeled hUCV-MSCs were detected in different parts of the ovarian tissue such as the medulla, especially near the medullary veins, cortex, between the follicles, and germinal epithelium of the CTX + hUCV-MSCs group, but these cells were not found inside the follicles and oocytes Fig. 4A) and in the tissue sections of other groups (Fig. 4B). The mean number of hUCV-MSCs was 35 ± 4.1 in the total ovarian region, 20 ± 2.5 in the medulla, 11.3 ± 2.8 in the cortex, and 5.5 ± 1 in the epithelium. The mean number of the fluorescent cells in the medullary region was significantly higher than the cortex and germinal epithelium (*p* < 0.001). Also, the mean number of CM-DiI-labeled hUCV-MSCs in the cortex was significantly higher than the germinal epithelium (*p* = 0.01), as shown in Figure 4C. The percentage of the fluorescent cells in the medulla, cortex, and epithelium were 57.1%, 32.2%, and 15%, respectively.

### Detection of human MTCYB gene in the ovarian tissue

**Fig. 5:**
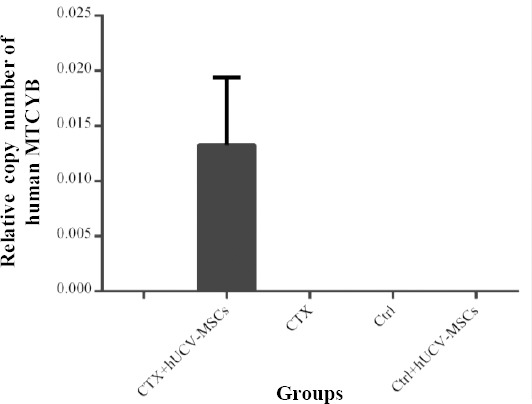
Detection of the human MTCYB gene in the mice ovarian tissues after the administration of human umbilical cord vein mesenchymal stem cells (hUCV-MSCs). Human MTCYB gene was detected only in the mice receiving cyclophosphamide (CTX) and hUCV-MSCs (CTX + hUCV-MSCs group), but not in the mice groups receiving CTX (CTX), PBS (Ctrl), and PBS + hUCV-MSCs (Ctrl + hUCV-MSCs).

Real-time PCR was used for each tissue sample to evaluate the presence of human MTCYB gene. Mouse GAPDH was used as a reference gene to normalize the results. CT, ΔCT, and the relative copy number of the samples were also calculated. Human MTCYB gene was detected in the CTX + hUCV-MSCs group, but not in other groups (Fig. 5).

## DISCUSSION

In the present study, the CTX-induced POF model was used to assess the distribution of the hUCV-MSCs in the mice ovarian tissues. POF model was confirmed by observing degenerative changes in the ovarian tissues. This model was in accordance with the previous studies[11,23]. MSCs-based cell therapy is an important research field in regenerative medicine for the treatment of various diseases. MSCs can migrate to damaged tissues and repair them by modulating the immune system and secreting growth factors[24,25].

The healing effects of MSCs on injured ovarian tissues have been reported in the POF animal models[26,27]. UC-MSCs are preferred for clinical applications because of their accessibility and poor immunogenic properties, which are attributed to their low expression of major histocompatibility complex I and the absence of major histocompatibility complex II. Moreover, UC-MSCs can be easily isolated and expanded *in vitro*, and they have little ethical issues as compared to other types of MSCs[28]. Wang *et al*.[11] have demonstrated that hUC-MSCs could recover ovarian structure and improve ovarian function injured by CTX in the mice. Ghadami *et al*.[29] have shown that i.v.-injected BM-MSCs are able to increase the FSH receptors, resume estrogen hormone production, and restore folliculogenesis in POF mice. In addition, adipose-derived MSCs and amniotic fluid MSCs have been referred to as therapeutic agents for chemotherapy-induced ovarian damage[18,30].

In the current study, CM-DiI-labeled hUCV-MSCs were injected into the POF mice. Red fluorescent cells were observed in the ovarian sections of the CTX-injured mice receiving hUCV-MSCs. Also, human MTCYB gene was detected in the ovarian tissues of CTX + hUCV-MSCs group, but not in other groups. Therefore, our data confirmed the engraftment of hUCV-MSCs in the ovaries of the CTX-injured mice. Wang *et al*.[11] have detected CM-DiI-labeled hUCV-MSCs in the POF mice ovaries one week after the i.v. injection. It seems that the migration of MSCs toward injured ovaries in the POF mice was due to the degenerative changes after CTX administration. Studies have confirmed these degenerative changes in POF models[18,29]. MSCs could migrate toward the injured tissues due to increased chemokines in the damaged and inflamed tissues. MSCs express molecules that mediate migration to the target tissues such as CXCR4 (C-X-C chemokine receptor type 4), CXCR7, and integrins. CXCR4 is one of the most known chemokine receptors involved in the migration of MSCs. The stromal-derived factor 1 (SDF-1) is the CXCR4 ligand increased in ischemic and injured tissues[24,31]. It appears that CXCR4/SDF-1 axis has an important role in the migration of the MSCs to the CTX-injured ovaries[18].

In our study, hUCV-MSCs were not distributed equally in different parts of the ovarian tissue. Fluorescent cells were mostly observed in the medulla compared with the cortex and the germinal epithelium. Also, most of the cells migrated to the ovarian stromal region. Fewer hUCV-MSCs reached the cortex, and smaller numbers of them were found in the germinal epithelium tissue. Moreover, few hUCV-MSCs were observed in the interface between primordial and primary follicles. hUCV-MSCs were not detected within primary follicles or even adult follicles. Wang *et al*.[11] have detected the MSCs in the ovarian stromal tissue, which is consistent with our findings. Sun *et al*.[23] have found labeled adipose-derived MSCs in the ovarian stromal tissue after i.v. injection, but not inside the follicles or oocytes. On the other hand, Liu *et al*.[18] have detected green fluorescent protein-labeled BM-MSCs in the ovarian tissue of the cisplatin-induced POF mice model after MSCs transplantation. The majority of MSCs were replaced in the medulla, and fewer cells were observed in the cortical region of the ovarian tissue. The cells were absent in the follicle and corpus luteum. Additionally, MSCs were mainly distributed along the blood vessels[18]. It has been shown that some of the MSCs are found in the cortical stromal region in the space between the follicles, and few numbers of cells are inside the follicles[32].

The therapeutic effect of MSCs can be related to more replacement of these cells in the stromal region of the injured ovaries. The effects of MSCs may be due to their active paracrine function by secreting growth factors to repair damaged follicles. Also, engrafted MSCs around the blood vessels can induce angiogenesis by secreting angiogenic growth factors[33,34]. The fact that MSCs were not seen in the follicles or oocytes could indicate that MSCs do not differentiate into follicular cells, and they indirectly affect follicular regeneration[33]. The most well-known growth factors secreted by MSCs are vascular endothelial growth factor, hepatocyte growth factor, and insulin-like growth factor[33]. Vascular endothelial growth factor is of paramount importance as it affects the growth of blood vessels in the granulosa follicular cell layers, and it prevents apoptosis[34].

Quantitative distribution of MSCs in different regions of the ovarian tissue in the POF mouse model has not been reported. We observed that the number of the CM-DiI-labeled hUCV-MSCs in ovarian medulla was greater than that of the ovarian cortex and germinal epithelium. Besides, the mean number of the labeled hUCV-MSCs in the cortex region was significantly higher than the germinal epithelium. hUCV-MSCs migration towards the medulla may be due to the fact that it is mostly composed of stromal tissue rather than the cortex and germinal epithelium, and also that medulla is rich in blood vessels. The stromal region of the ovarian tissue is the source of SDF-1 during injury and inflammation; as a result, it increases the migration of MSCs to this region[18]. Meanwhile, physical barriers in ovarian tissue can also be a limiting factor for the homing of MSCs in a particular region of the tissue. For instance, the reason for the small numbers of MSCs engraftments in the ovarian germinal epithelium could be related to the preventing effects of physical barriers such as basal membranes and cell-cell junction complexes[35]. hUCV-MSCs were not observed in the follicles, cumulus cells, or oocytes. Tight junctions between the follicular cells may prevent the cells from entering the follicles. Furthermore, intracellular connections and gap junctions in both inner and outer theca cell layers in the mature follicles could be an obstacle to MSCs homing. In addition, the presence of a barrier around the follicle like the basement membrane can prevent MSCs from entering the follicles[36].

The findings of the present study show that hUCV-MSCs home in the CTX-injured mice ovaries with more engraftment in the medulla and stromal region, and a small number of hUCV-MSCs engraft in the ovarian cortex and germinal epithelium.
